# Stability Studies of a Tetraethyl Orthosilicate-Based Thixotropic Drug Delivery System

**DOI:** 10.3390/pharmaceutics16111392

**Published:** 2024-10-29

**Authors:** Emma Barrett-Catton, Elizabeth M. Arrigali, Bogdan A. Serban, Kolton C. Sandau, Monica A. Serban

**Affiliations:** 1 Department of Biomedical and Pharmaceutical Sciences, University of Montana, Missoula, MT 59812, USA; 2 Montana Biotechnology Center (BIOTECH), University of Montana, Missoula, MT 59812, USA

**Keywords:** thixotropic, shelf life, drug delivery system

## Abstract

**Background/Objectives:** This study assessed the effects of formulation components on the long-term stability of a previously described tetraethyl orthosilicate-based drug delivery system. Early stability studies of a product concept are crucial not only to provide information on the overall system stability and individual components’ contributions to it, but also to identify opportunities for dosage form optimization and to define its use case. **Methods:** We assessed the time-dependent thixogel properties—specifically, mechanical strength, thixotropy, release of model drug, and dry substance—in both real-time and accelerated shelf-life determination set-ups. **Results:** Our findings indicate that the concentration and molecular weight of hyaluronic acid, one of the main constituents of the investigated thixotropic systems, are key determinants of formulation stability. We further showed that changes in both of these parameters reflect on the drug release properties and stiffness of the formulation and could inform subsequent product development based on several use cases. **Conclusions:** Overall, this study provides an understanding of some key factors that would need to be considered prior to and in the final product development process of thixogels in preparation for commercialization.

## 1. Introduction

Otitis externa, or outer ear infection, is a prevalent condition that affects both humans and animals [1,2,3,4,5]. Commonly, otitis externa is treated by using antibiotic ear drops, which must be applied multiple times a day for several consecutive days over the course of the infection [6,7,8,9]. However, this treatment regimen is associated with poor patient compliance, which typically leads to continued or severe cases of infection [7,8,9]. To address this issue, several single-application therapeutic alternatives have been developed. However, the few existing single-application treatments approved for use in humans and animals have several disadvantages, including cold-chain dependence, as well as formulations that are difficult to handle and apply [10,11,12].

The development and characterization of a tetraethyl orthosilicate-based hydrogel for use as a single-application treatment for otitis externa has been formerly reported by our group [13,14,15,16]. The design of this material was specifically tailored to overcome issues such as the need for a cold chain, as well as cumbersome handling and application. Additionally, this hydrogel was formulated to exhibit thixotropy, a rheological property that enables it to become liquid-like under stress (e.g., shear) and then return to its original gel-like state when the stress is removed [17,18]. This property allows it to be deployed as a liquid to the site of infection—for example, through a cannulated syringe—where it will then rapidly gel under static/stress-free conditions and locally release the infection-fighting therapeutic agent. Throughout this paper, we will therefore refer to the tetraethyl orthosilicate-based hydrogel as a thixogel.

To advance the commercialization of this thixogel, we previously assessed its scalability and manufacturability [16]. In the current study, we sought to understand the effects of the formulation components on the shelf life (or long-term stability) of the thixogel. The shelf life of a product is considered to be reflective of the extent to which the product remains within the acceptable specification range established during its development/manufacturing process. While the shelf life of any final product in the form of a prepackaged thixogel/drug combination would need to be addressed individually because the nature of the drug used is expected to impact the loaded gel’s shelf life, with this study we aimed to understand the correlation between the thixogel’s storage time, temperature, and formulation. Our thixogel is made of three main components: hydrolyzed tetraethyl orthosilicate (hTEOS), sodium hyaluronate (HA), and ammonium hydroxide (NH_4_OH) [16]. Both tetraethyl orthosilicate and sodium hyaluronate (HA) are commonly used in medical and cosmetic formulations, making manufacturing with these materials more streamlined as they are already established materials [19,20]. However, the shelf life of HA-based biomaterials is highly variable. For instance, the shelf life of HA-based dermal fillers can range from 9 months to 3 years [21]. Therefore, in this study, we tested the effects of HA concentration and molecular weight on the time-dependent thixogel properties; specifically, we assessed mechanical strength, thixotropy, release of model drug, and dry substance, in both real-time and accelerated shelf-life determination set-ups. Collectively, through this study, the evaluation of the formulation’s shelf life was intended not only to provide crucial information on stability but also to identify opportunities for dosage form optimization and to define its use case.

## 2. Materials and Methods

### 2.1. Materials

The following materials were used for this study: tetraethyl orthosilicate [(TEOS), Alpha Aesar/Thermo Scientific, Waltham, MA, USA], sodium hyaluronate [(HA, 5 kDa), Lifecore Biomedical, Chaska, MN, USA], sodium hyaluronate [(HA, 13.8 kDa), HTL Biosciences, Javene, France], acetic acid [(HOAc), EMD Millipore, Billerica, MA, USA], ammonium hydroxide [(NH_4_OH), Fisher Chemical, Fair Lawn, NJ, USA], phosphate-buffered saline [(PBS), Corning Life Sciences, Durham, NC, USA], and fluorescein (Fluka Analytical, owned by Sigma-Aldrich, St. Louis, MO, USA).

### 2.2. Thixogel Preparation

To prepare thixogels TEOS was hydrolyzed with 0.15 M HOAc for 1.5 h at a 1:9 *v*/*v* ratio. The hydrolyzed material (hTEOS) was then formulated with HA solutions (10%, 0.5%, or 0.1% *w*/*v* in 1X PBS) at a 1:2 *v*/*v* ratio. HA 5 kDa was tested at all concentrations, while HA 13.8 kDa was tested at 10% *w*/*v*. The mixtures were vortexed, their pH was adjusted to ~7.65 with 1.5 N NH_4_OH and were left undisturbed overnight at room temperature to gel.

### 2.3. Shelf Life Studies

The shelf life for all of the thixogel variations was tested at room temperature. Additionally, accelerated aging/shelf life experiments at 37 °C were also carried out for thixogels made with 0.1% *w*/*v* and 0.5% *w*/*v* 5 kDa HA, as well as 10% *w*/*v* 13.8 kDa HA, using the accelerated aging factor to determine the equivalent room temperature aging [22]:

$$AAF=2^{(T_{aa}-T_{rt})/10}$$

where *AAF* is the accelerated aging factor, *T_aa_* is the accelerated aging temperature, and *T_rt_* is the room temperature. For the accelerated stability studies, we chose temperatures of 37 °C for thixogels to also coincide with the physiologically representative drug release conditions. For HA solutions, accelerated degradation was conducted at 40 °C to speed up the acquisition of experimental data while eliminating the possibility of any artefactual higher-temperature-induced sample degradation. Using 22 °C for room temperature, the accelerated aging factor at 37 °C was 2.82, and the accelerated aging factor at 40 °C was 3.48. These estimations mean that aging the thixogels for one month at 37 °C is the equivalent of aging the thixogels at room temperature for approximately 2.82 months, while aging the thixogels for one month at 40 °C is the equivalent of aging the thixogels at room temperature for approximately 3.48 months. It is important to note that these are approximations and are not perfect equivalents of the aging of HA-based products.

### 2.4. Drug Release Studies

These studies were conducted by using fluorescein as a drug model for ease of monitoring. Fluorescein stock solution aliquots (20 µL of 10 mg/mL fluorescein in 0.15 M NH_4_OH) were combined with 1980 µL hTEOS/HA formulations prepared as described above. The mixtures were allowed to gel overnight at room temperature then were washed with PBS to remove ethanol. Thixogels were then covered with a 2 mL volume of PBS and the vials were placed at 37 °C with no shaking. Aliquots of 100 µL PBS from each vial were collected at 24 h timepoints. The amount of fluorescein released was determined based on absorbance values at 450 nm, collected with a BioTek Cytation 5 plater reader (Winooski, VT, USA) using Gen5 microplate Reader and Imager software version 3.11. After each collection, for each sample the PBS was discarded and replaced with fresh aliquots daily. 

### 2.5. Rheological Characterization

A hybrid Discovery HR-2 Rheometer/Dynamic Mechanical Analyzer (TA Instruments, New Castle, DE, USA) was used for these analyses with TA Instruments TRIOS software version 5.1.1.46572. Characterizations were performed within the materials’ pseudolinear viscoelastic range by using a 1.00 mm gap, at room temperature. Each test included an initial conditioning step to establish an axial force between 0.1 and 0.3 N. Thixotropy analyses were performed for 3 cycles with a 30 s rest between each cycle, with oscillatory strain that used a 20 mm parallel-plate geometry, a strain range of 1–100% and an angular frequency of 10 rad/s. The storage moduli of thixogels were determined via oscillatory frequency sweeps from 100 Hz to 0.1 Hz with 50 Pa stress. The storage moduli were compared to specifications from 720 to 1440 Pa previously determined by our group for the standard formulations containing 10% *w*/*v* 5 kDa HA, calculated by determining the mean and standard deviation of a large sample size of thixogels. The specifications were defined as the mean ± 2 standard deviations.

### 2.6. Dry Substance Characterization

An MJ33 moisture analyzer (Mettler Toledo, Columbus, OH, USA) was used to analyze the dry substance content of thixogels. For this, samples were placed in the analyzer and evenly distributed with a spatula, then dried at 104 °C until no weight variations were detected. The percentage of dry substance per thixogel was then calculated as the ratio of the final weight recorded to the initial weight loaded.

### 2.7. Spectrophotometry

Solutions of 10% *w*/*v* 5 kDa and 13.8 kDa HA in 1X PBS were prepared and stored at 40 °C for three months. Additionally, fresh solutions of 5 kDa and 13.8 kDa HA were prepared at the time of testing. All four solutions were diluted to 1.5 mg/mL. An absorption scan from 200 to 800 nm was run on all diluted solutions using a Cary Series UV–Vis Spectrophotometer (Agilent Technologies, Santa Clara, CA, USA) with Cary-WinUV software version 4.20.

### 2.8. Statistical Analyses

Two-group comparisons were conducted with two-tailed Student’s *t*-tests with α = 0.05. For groups larger than two we employed one-way ANOVA analyses with either Tukey’s multiple comparisons (all groups compared to one another) or Dunnett’s multiple comparisons (all groups compared to a control group) with α = 0.05. For the drug release studies, based on the number of groups we employed either two-tailed Student’s *t*-tests or one-way ANOVA analyses. The software used for statistical analyses was Prism version 10.1.1. The error bars in all figures correspond to standard deviation values.

## 3. Results

### 3.1. Stability of Standard Thixogel Formulation

The time-dependent stability of our original, standard thixogel formulation (10% *w*/*v* 5 kDa HA) was first determined. For this, we measured the drug release, rheological properties, and dry substance of the thixogels after 0, 1, 2, 3, 4, and 5 months of room-temperature storage, using a total of three samples per timepoint.

**Figure 1:**
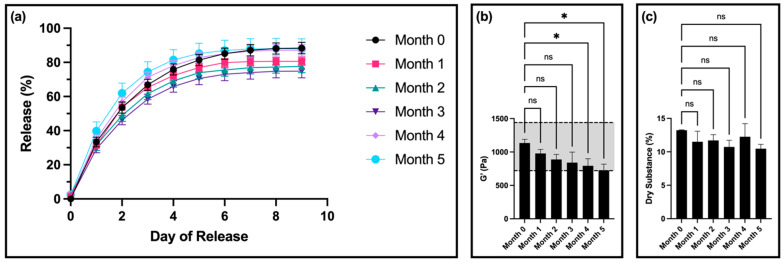
Stability of standard 10% *w*/*v* 5 kDa HA thixogel formulations: (**a**) Drug release profiles of hydrogels at different stability points (real time, 0, 1, 2, 3, 4, and 5 months). (**b**) Storage modulus values for the original thixogel formulation stored over time. (**c**) Dry substance percentage for the original thixogel formulation stored over time. Figures display means and standard deviations (N = 3). For the rheological data, the grayed-out area represents the previously determined specifications for storage modulus from 720 to 1440 Pa. Means were compared with one-way ANOVA, with * indicating *p* < 0.05, and ns indicating insignificance (for figure clarity, these symbols are not used for the drug release data).

For drug release, there was no significant difference in the release profiles for samples from months 1, 4, and 5 compared to month 0 (Figure 1a). Interestingly, samples from months 2 and 3 had a statistically significantly lower amount of fluorescein released compared to month 0.

After 4 months, compared to the control, the storage modulus of the gels was significantly decreased but remained within the set specification. However, at 5 months, the storage modulus was outside of the specification window (Figure 1b). The thixotropic behavior of the materials remained unaltered (Figure S1).

**Figure 2:**
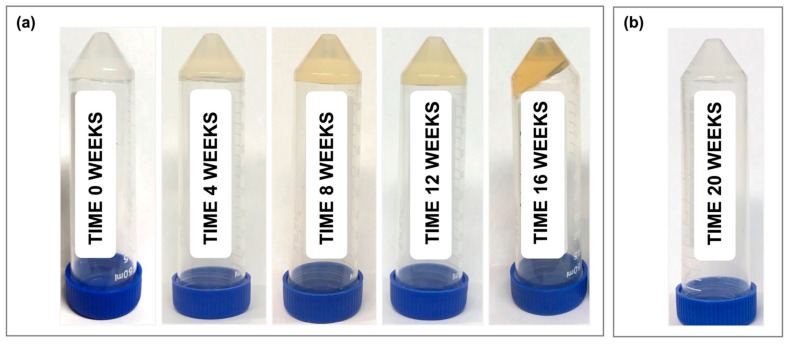
Visual appearance of thixogels over time, aged at room temperature: (**a**) 10% 5 kDa HA thixogel formulation for 0, 1, 2, 3, and 4 months, from left to right; thixogels aged for 5 months at room temperature could no longer be held upside-down and, therefore, are not pictured here; (**b**) thixogel formulation with no HA in its composition (TEOS-only), aged for 5 months at room temperature.

In terms of dry substance content, there was no significant difference between samples for any of the timepoints (Figure 1c). Furthermore, all samples showed a visible color change, transitioning from clear (month 0) to a light yellow color at month 1, and progressing to a dark orange by month 4 (Figure 2a). At 5 months, the room-temperature, standard thixogel formulation samples transitioned to a very soft gel/liquid-like state. Importantly, control TEOS-only thixogels (with no HA in the formulation) equivalently aged for 5 months at room temperature maintained their physical appearance (Figure 2b).

### 3.2. Effects of HA Concentration on Thixogel Stability

Based on our findings above, which suggest that the shelf life of thixogels is primarily dictated by their HA component, we sought to understand the effect of the HA concentration on formulation stability. Specifically, we prepared thixogels containing 0.5% and 0.1% *w*/*v* HA (instead of the 10% *w*/*v* used in the standard formulation) and measured the formulations’ properties after 0, 1, and 3 months of room-temperature storage, along with 1, 2, and 3 months of storage at 37 °C. The accelerated aging conditions correspond to approximate real times of 3, 5.5, and 8.5 months.

**Figure 3:**
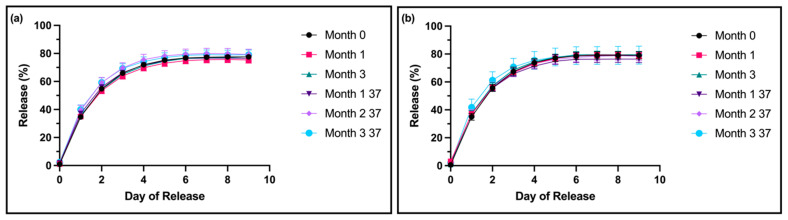
Drug release profiles of low-concentration thixogel formulations: (**a**) The fluorescein release profiles of 0.5% *w*/*v* 5 kDa HA thixogel formulations at various timepoints; (**b**) The fluorescein release profiles of 0.1% *w*/*v* 5 kDa HA thixogel formulations. For both HA concentrations, samples were tested after 0, 1, and 3 months of storage at room temperature and 1, 2, and 3 months at 37 °C, with N = 3 per timepoint. Some error bars are not visible due to the plot symbols. Drug release from thixogels at different timepoints were compared to month 0 with one-way ANOVA with no statistically significant differences identified.

For both the 0.5% *w*/*v* 5 kDa HA and the 0.1% *w*/*v* 5 kDa HA formulations, no significant differences were noted between the drug release profiles of any test samples compared to the month-0 controls (Figure 3a,b). Compared to the standard formulation with 10% *w*/*v* HA 5 kDa, at month 0, both lower-HA formulations showed decreased drug release profiles starting at day 6.

**Figure 4:**
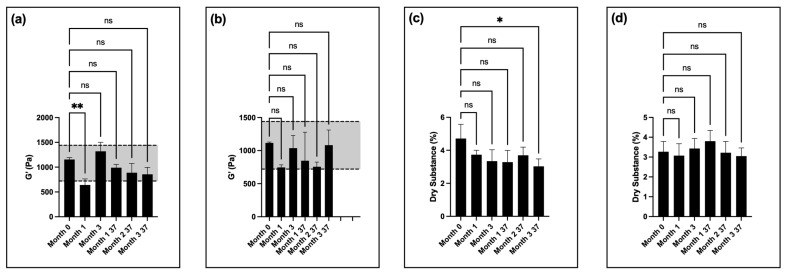
Time-dependent stability of low-HA-concentration thixogels: (**a**) Storage moduli for 0.5% *w*/*v* 5 kDa HA thixogels over time. (**b**) Storage moduli for 0.1% *w*/*v* 5 kDa HA thixogels over time. (**c**) Dry substance percentage for 0.5% *w*/*v* 5 kDa HA thixogels over time. (**d**) Dry substance percentage for 0.1% *w*/*v* 5 kDa HA thixogels over time. Figures display means and standard deviations (N = 3). For the storage modulus data, the grayed-out area represents the previously determined specifications for the standard formulation (720 to 1440 Pa). Statistical analyses were performed with one-way ANOVA, with * indicating *p* < 0.05, ** indicating *p* < 0.01, and ns indicating insignificance.

For the storage moduli of the low-HA formulations, the samples stayed within the specifications for all timepoints and test conditions, other than month 1 for 0.5% *w*/*v* HA thixogels at room temperature (Figure 4a,b). Three samples were tested here for each timepoint. Similar to the standard formulation, the thixotropic behavior of the materials was unaffected by the observed changes in G′ values, even at the final timepoints assessed (Figures S2 and S3).

The amount of dry substance in the thixogels was consistent for all timepoints, other than month 3 of accelerated aging for 0.5% *w*/*v* HA thixogels (Figure 4c,d), which was most likely indicative of some evaporation.

In terms of physical appearance, the 0.5% *w*/*v* HA thixogels began to display a light yellow coloration only after 3 months at 37 °C (equivalent of ~8.5 months’ real-time stability), but there was no visibly detectable color change for the 0.1% *w*/*v* HA thixogels at any of the timepoints tested.

### 3.3. Effects of HA Molecular Weight on Thixogel Stability

With an understanding of the effect of HA concentration on time-dependent thixogel stability, we next investigated the effect of HA MW on thixogel shelf life. For this, we formulated thixogels with 10% *w*/*v* 13.8 kDa HA and measured their properties after 0, 1, and 3 months of room-temperature storage, as well as after 1, 2, and 3 months of storage at 37 °C. Other higher-molecular-weight HA formulations were not explored here, based on our previous findings that higher-molecular-weight polymers impair thixogel formation, along with high-MW HA solubility issues.

**Figure 5:**
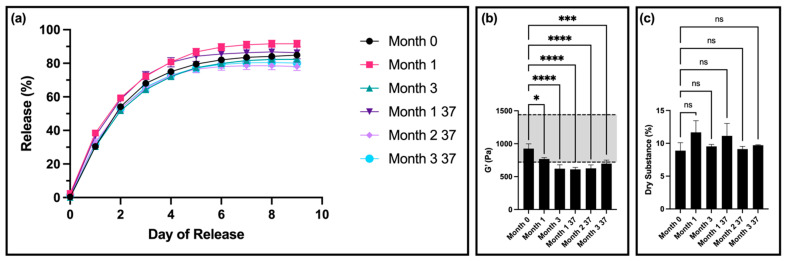
Stability of 10% *w*/*v* 13.8 kDa HA thixogel formulations: (**a**) Drug release profiles of hydrogels at different stability points (0, 1, and 3 months of storage at room temperature and 1, 2, and 3 months at 37 °C). (**b**) Storage moduli for 13.8 kDa HA thixogels over time. (**c**) Dry substance percentage for 13.8 kDa HA thixogels over time. Figures display means and standard deviations (N = 3). For the storage modulus data, the grayed-out area represents the previously determined specifications for the standard formulation (720 to 1440 Pa). Means were compared with one-way ANOVA, with * indicating *p* < 0.05, *** indicating *p* < 0.001, **** indicating *p* < 0.0001 (for figure clarity, these symbols are not used for the drug release data), and ns indicating insignificance. Some error bars are not visible due to plot symbols.

Thixogel samples stored at room temperature for one month had significant differences in release compared to month 0 on most days (Figure 5a). Thixogels stored at 37 °C for one month (corresponding to ~3 months of real time) had significant differences in release compared to month 0 on days 1 through 4 (Figure 5a). Thixogels stored at 37 °C for two months (corresponding to ~5.5 months of real time) had significant differences in release compared to month 0 on days 8 and 9 (Figure 5a). There were no significant differences in release profiles of thixogels stored for three months at room temperature and 37 °C (~8.5 months real time) compared to month 0 (Figure 5a).

The rheological data revealed significant differences in the storage moduli of the samples at all timepoints compared to month 0, with the stiffness values falling below the low end of the specifications at month 3 of room-temperature storage and month 1 of storage at 37 °C (equivalent to ~3 months of real time) (Figure 5b). It is important to note here that the starting G′ values of the 13.8 kDa thixogels were significantly lower than those of equivalent concentrations of 5 kDa thixogels (G′ = 1135 ± 115 Pa for the standard formulation versus G′ = 925 ± 42 Pa for the 13.8 kDa formulation). This is in line with our previous observations that higher-MW polymers lead to the disruption of the thixotropic network and result in softer gels when gelation occurs. The 13.8 kDa material formulations did exhibit thixotropic properties even at the final timepoint evaluated (Figure S4).

In terms of dry substance content, there were no significant differences between the thixogels at any of the timepoints (Figure 5c). Regarding the visual appearance of the samples, solutions of both 5 kDa and 13.8 kDa in PBS at 40 °C for 3.5 months (12-month room-temperature equivalent) exhibited a color change, with the 5 kDa solution turning a darker yellow compared to the 13.8 kDa HA solution.

### 3.4. Spectrophotometric Assessments of MW-Dependent HA Stability

The results described above clearly indicate a direct correlation between the observed changes in the formulations’ properties and HA concentration and MW. To better understand these processes, we sought to investigate the absorbance spectra of HA-only aqueous solutions under real-time and accelerated aging conditions similar to those used for the thixogels.

**Figure 6:**
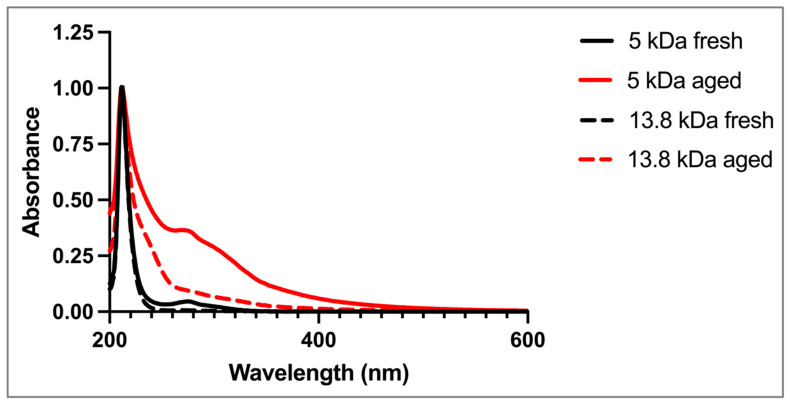
Spectrophotometric analyses of HA stability: UV–Vis absorbance scans from 200 nm to 800 nm of fresh and aged (40 °C for 3.5 months, equivalent of ~12 months at room temperature) 5 kDa and 13.8 kDa HA solutions. The peak at ~280 nm indicates HA depolymerization.

An initial observation was that the fresh HA solutions (10% *w*/*v*, 5 kDa and 13.8 kDa) were clear, while aged HA solutions were yellow in color, similar to the changes in physical appearance observed in the 10% *w*/*v* HA thixogels. The spectrophotometric analysis of all solutions, fresh and aged, displayed a characteristic peak at 212 nm, corresponding to intrinsic C=O bonds of HA (Figure 6) [23,24,25]. The fresh 5 kDa solution exhibited a peak at ~280 nm that was not observed in the fresh 13.8 kDa HA solutions. The spectra of the aged 5 kDa and 13.8 kDa HA solutions both showed the ~280 nm peak, along with increased absorbance in the 240 nm region and the 400–550 nm region.

## 4. Discussion

The work presented herein describes our approach to understanding the time-dependent stability of thixogels and the factors that influence it. The time-dependent stability or the shelf life of a formulation is an important parameter of any product. Understanding it early on in the development process is crucial for positioning a product for rapid advancement to commercialization. The shelf life informs us not only on the formulation’s stability but also on the type of dosage form it should be developed into for productization. Therefore, the first aspect that we investigated in this study was the stability of our standard thixogel formulation containing 10% *w*/*v* 5 kDa HA, considering that most commercial products based on HA contain high-MW and/or crosslinked versions of it.

Our thixogels contained low-MW HA, which was selected based on its ability to increase the biological compatibility of the thixogel as well as its ability to integrate well with the hTEOS thixotropic network [13,14,15]. Interestingly, we found that HA was the main parameter that dictated the thixogels’ stability. The standard formulation showed no significant difference in the drug release profiles for some of the timepoints, especially later ones (months 4 and 5), compared to month 0. However, changes were detected for some of the intermediate timepoints (month 2 and month 3), potentially indicating the occurrence of dynamic, time-dependent processes that affect the thixogels’ structure and interaction with the model drug tested. The decrease in storage modulus is also indicative of a decrease in the amount of structure present in the thixogel. The visually detectable color changes also suggested that chemical and/or physical processes were happening over time. Considering the simple composition of the thixogels comprising hTEOS and HA, along with the lack of visually detectable changes in aged hTEOS/control thixogels, it became clear that the HA component was likely catalyzing those changes. Our results indicate that, relative to the existing set specifications, the shelf life of the standard thixogel formulation was around 4 months, but visually detectable changes were apparent within one month of preparation. From a product development and commercialization perspective, these data suggest that the standard formulation would be primarily suitable for compounding applications, where it would be used immediately or shortly after preparation.

To further test the role of HA in thixogel stability, we assessed the effects of both HA concentration and MW. We found that a decrease in HA concentration directly translated to an increase in thixogel stability, with the 0.1% *w*/*v* 5 kDa HA thixogel showing an ~8.5-month real-time shelf life. The drug release profiles of the low-HA-concentration thixogels were decreased compared to the standard formulation, indicating stronger interactions between the model drug tested and the hTEOS network. While experimentally significant, it is unclear whether, from a clinical and therapeutic perspective, the differences in the amount of drug released by the low-HA thixogel formulations versus the standard one would be of importance. Regardless of this study, this aspect will need to be subsequently tested in preclinical studies with actual therapeutically relevant drug and final thixogel formulations/products. The storage moduli of these low-HA-concentration formulations showed more stability, except for the month-1 0.5% *w*/*v* HA thixogels at room temperature, most likely reflective of HA-associated dynamic processes, as indicated previously. It is important to note here that the thixogel specifications that were used as benchmark for these stability studies—specifically, the thixogel storage moduli—were set based on the standard formulation [16]. Outside of the specification values (<720 Pa) however, our findings do not indicate that such formulations would be incompatible with the intended application, considering that materials with G′ values between 0.01 and 1 Pa are categorized as soft hydrogels [26]. From a manufacturing and product cost perspective, the productization of the low-HA thixogel formulation has clear economic advantages. Overall, thixogels with reduced HA concentrations appear to have increased stability compared to the standard formulation, with visual color changes only observed after 8.5 months at room temperature equivalent for the 0.5% *w*/*v* HA-containing formulations, while the G′ values stayed within the specifications. These results indicate that such formulations might be a viable option for the development of off-the-shelf products, with ~1-year-long shelf lives.

The study of the effect of HA MW on thixogel stability presented additional stability optimization results. Thixogels prepared using 10% *w*/*v* 13.8 kDa HA had a more subtle color change compared to their counterparts prepared with 5 kDa HA. Overall, the time-dependent drug release profiles of the 13.8 kDa HA formulations fell within the range of those observed for the 10% 5 kDa HA formulations, and as indicated above, the therapeutic relevance of these release profiles will have to be determined with actual, application-specific drugs in future studies. The storage moduli of the 13.8 kDa HA thixogels fell below the specifications after two months of room-temperature storage, and after one month of storage at 37 °C (equivalent of ~3 months of real time). However, the later-timepoint values remained fairly constant throughout the evaluation period, in the ~500 Pa range, indicative of stability compared to the standard formulation, where the G′ values displayed a steady decreasing trend. Together, these data indicate that 13.8 kDa thixogels could be another viable alternative for the development of off-the-shelf products, with ~1-year-long shelf lives. All thixogel formulations assessed in this study maintained their thixotropic properties and showed no notable changes in this property [16].

With a clear understanding that the visually observed color changes and overall time-dependent changes in thixogel properties were HA-related, the time-dependent behavior of HA-only fresh and aged solutions was evaluated spectrophotometrically. Our first observation here was that both the 5 kDa and 13.8 kDa HA solutions in PBS at 40 °C, aged for 3.5 months (12-month room-temperature equivalent), exhibited a clear color change, with the 5 kDa solution showing more intense coloration compared to its 13.8 kDa counterpart. The spectrophotometric profiles of the fresh and aged HA solutions offered additional insights into the time-dependent physicochemical changes that the glycosaminoglycan molecules were undergoing. Specifically, the 212 nm peak is common to the double bonds of C=O keto- or carboxy- functionalities intrinsic to the macromolecules [25,27]. The fresh 5 kDa HA sample showed a peak at ~280 nm, which was not present in the fresh 13.8 kDa sample. However, this peak was present in both aged samples, and it was significantly more prominent in the 5 kDa aged sample than in the 13.8 kDa aged sample. This peak has been associated with the presence of degradation-induced generation of new carbonyl and carboxyl groups in HA [24]. Both aged samples also showed an absorbance shoulder in the 240 nm range, indicative of unsaturated structures [24], as well as in the 400–550 nm region, consistent with the visually detected yellow/yellow–orange coloration at later storage timepoints. Collectively, the spectrophotometric analyses seem to confirm that HA undergoes chemical degradation, which seemed more pronounced in the lower-MW sample.

The actual mechanism of HA’s depolymerization is unclear; however, our data allowed us to hypothesize. HA is a polymeric glycosaminoglycan consisting of repeating units of glucuronic acid and N-acetyl glucosamine [28]. Commercially available HA samples are always polydisperse mixtures, with the predominant MW peak designated as the product’s MW. The 5 kDa HA sample was a polydisperse mixture of HA fragments with an MW of 4.5 ± 0.5 kDa and a polydispersity index of 1.3 ± 0.05 [16]. When the 5 kDa HA solution was dialyzed in a 3500 MWCO dialysis cassette, the aging-associated yellowing process of the solution was significantly delayed compared to the non-dialyzed samples, suggesting that low-MW species are responsible for the overserved color changes. It is possible that these smaller HA oligomers undergo a Maillard reaction/Amadori rearrangement, commonly associated with the browning of sugar molecules. Maillard reactions involve interactions between amines and carbonyl functionalities to yield Amadori products [29]. Amadori rearrangements are typically base- or acid-catalyzed reactions between an amine and the hydroxyl group of a sugar, resulting in the isomerization of the sugar from an aldose to a ketose [30,31]. The reaction involves the formation of a Schiff base between the carbonyl and amine functionalities, followed by an Amadori rearrangement that yields Amadori rearrangement products, which are ultimately broken down into a wide variety of compounds, causing a yellow or brown color along with flavor and color changes [30,31]. Amadori rearrangement products have been shown to have an absorbance peak around 260 nm [32,33], and HA has previously been shown to undergo Amadori rearrangement [34,35,36]. All of these facts seem to support our hypothesis that such reactions occurred in our HA-containing samples. However, these reactions are typically accelerated at higher temperatures [31] and, as mentioned previously, acid/base-catalyzed. Our attempts to decrease the HA solutions’ yellowing by decreasing the temperature (4 °C versus room temperature versus 37 °C) and using a buffered system did not yield drastically improved results, and further investigation of these reactions was beyond the scope of this study.

We previously described the mechanism of thixogel formation, which is based on the colloidal aggregation of TEOS nanoparticles dominated by short-range van der Waals forces and surface charge interactions [13]. We believe that the degradation of the HA component of thixogels potentially interferes with these physical interactions between the TEOS nanoparticles and impacts the material’s rheological parameters. This rationale could also explain the unusual time-dependent trends observed in the G′ values for the 0.1% *w*/*v* and 0.5% *w*/*v* HA thixogels. Given that the starting HA concentration in these samples is lower than in the standard formulation, the interactions between the TEOS nanoparticles are more prevalent, and their disruption by HA degradation products is more readily evident at earlier timepoints. As we have previously shown that the nanoparticulate structure and stiffness of the thixogel undergo changes over time [13], it is possible that the thixotropic network is reconfigured at subsequent timepoints, as evidenced by a transient increase in G′ values, prior to undergoing a more typical time-dependent loss in material stiffness, similar to the standard formulation.

## 5. Conclusions

Herein, we have presented a study aimed at investigating the stability of HA/TEOS-based thixogels and factors that impact it, highlighting the versatility of this thixogel system for productization. Our data indicate that, due to HA-associated physicochemical changes, the standard formulation that contains 10% *w*/*v* 5 kDa HA would be primarily suitable for compounding applications, or for products with a ~4-month shelf life. We found that the use of higher-MW HA for formulations could increase the stability of the 10% HA formulations if desired. Lower-HA-concentration formulations might be a viable option for the development of off-the-shelf products with ~1-year-long shelf lives, most likely with a lower price point reflective of the decreased amount of HA needed for manufacturing. Overall, this study provides an understanding of some key factors that would need to be considered in the final product development process of thixogels, as well as for final product (thixogel + drug of interest) shelf-life determinations.

## Data Availability

The original contributions presented in the study are included in the article/Supplementary Material, further inquiries can be directed to the corresponding author.

## Supplementary Materials

The following supporting information can be downloaded at: https://www.mdpi.com/article/10.3390/pharmaceutics16111392/s1, Figure S1. Representative thixotropic profiles of gels obtained with 10% *w*/*v* 5 kDa HA formulations at time zero (left panel) and 5-month real-time aging (right panel). Figure S2. Representative thixotropic profiles of gels obtained with 0.5% *w*/*v* 5 kDa HA formulations at time zero (left panel) and 3-month accelerated aging (right panel). Figure S3. Representative thixotropic profiles of gels obtained with 0.1% *w*/*v* 5 kDa HA formulations at time zero (left panel) and 3-month accelerated aging (right panel). Figure S4. Representative thixotropic profiles of gels obtained with 10% *w*/*v* 13.8 kDa HA formulations at time zero (left panel) and 3-month accelerated aging (right panel).
