# Supplementary material for: Stability Studies of a Tetraethyl Orthosilicate-Based Thixotropic Drug Delivery System
Source: Pharmaceutics. 2024 Oct 29;16(11):1392. doi: 10.3390/pharmaceutics16111392 (PMC11597098; doi:10.3390/pharmaceutics16111392)
Supplement: Supplementary file 1 [file pharmaceutics-16-01392-s001.zip › pharmaceutics-3253461-supplementary.pdf]

Supplementary Materials for Stability Studies of a Tetraethyl Orthosilicate-Based Thixotropic Drug Delivery System

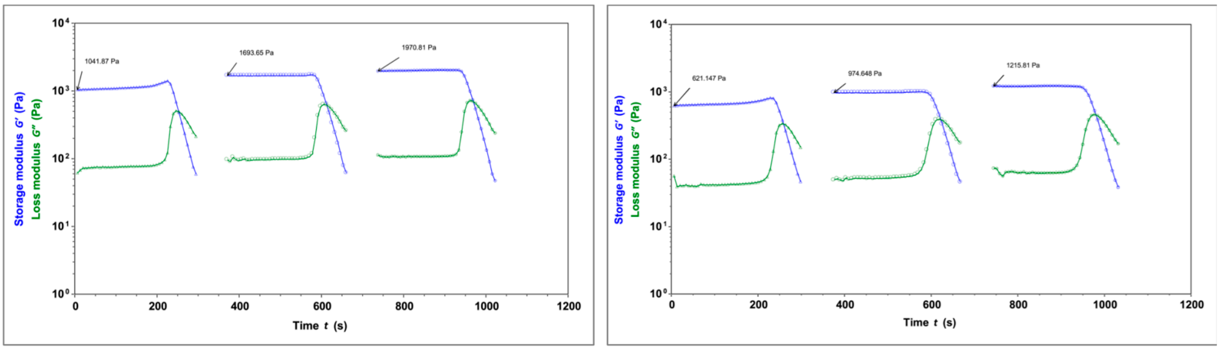

Figure S1. Representative thixotropic profiles of gels obtained with 10% w/v 5 kDa HA formulations at time zero (left panel) and 5-month real-time aging (right panel).

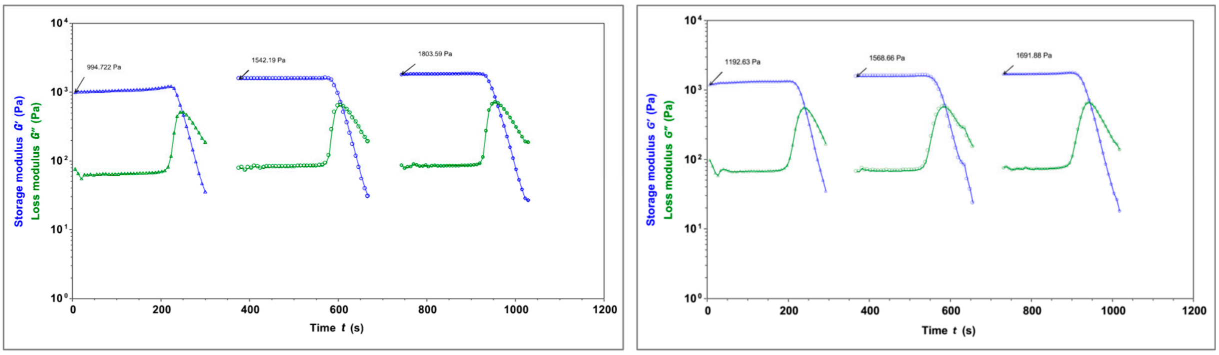

Figure S2. Representative thixotropic profiles of gels obtained with 0.5% w/v 5 kDa HA formulations at time zero (left panel) and 3-month accelerated aging (right panel).

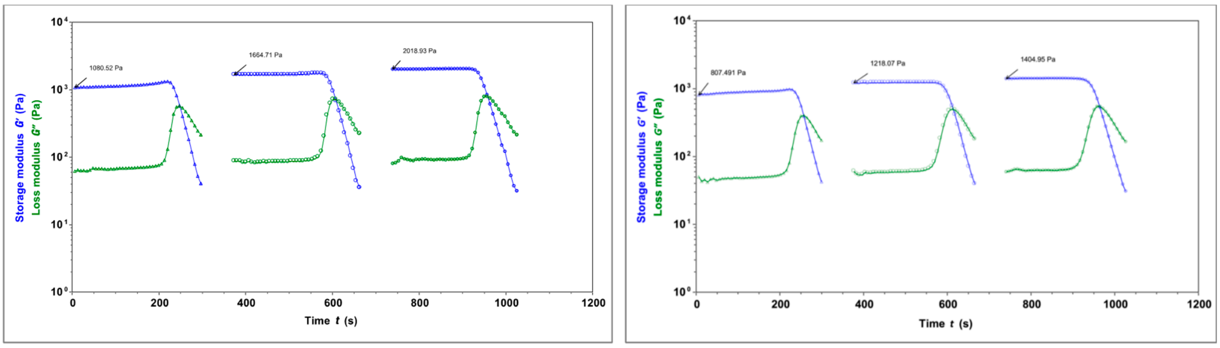

Figure S3. Representative thixotropic profiles of gels obtained with 0.1% w/v 5 kDa HA formulations at time zero (left panel) and 3-month accelerated aging (right panel).

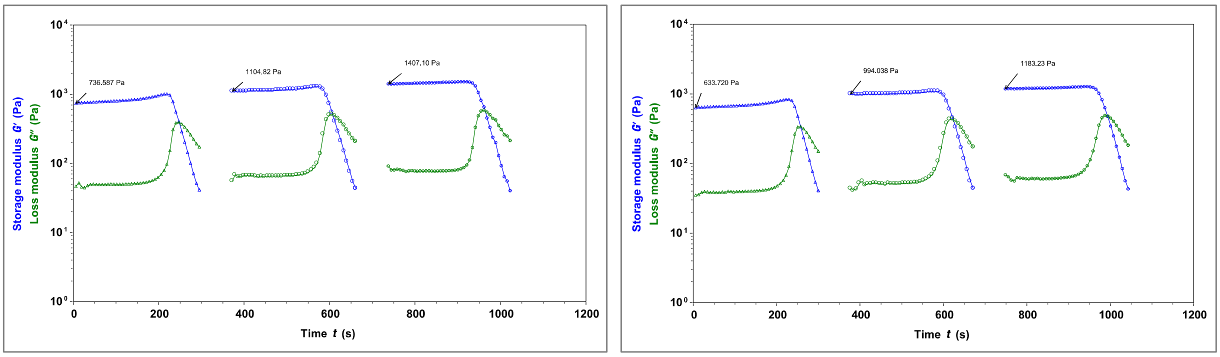

Figure S4. Representative thixotropic profiles of gels obtained with 10% w/v 13.8 kDa HA formulations at time zero (left panel) and 3-month accelerated aging (right panel).
